# Association between the Polymorphisms in Intercellular Adhesion Molecule-1 and the Risk of Coronary Atherosclerosis: A Case-Controlled Study

**DOI:** 10.1371/journal.pone.0109658

**Published:** 2014-10-13

**Authors:** Mao Yang, Zhenkun Fu, Qingjiang Zhang, Yu Xin, Yanjun Chen, Ye Tian

**Affiliations:** 1 Department of Cardiology, The Fourth Affiliated Hospital of Harbin Medical University, Harbin, China; 2 Department of Immunology, Harbin Medical University, Harbin, China; 3 Department of Cardiology, The First Affiliated Hospital of Harbin Medical University & Department of Pathophysiology, Harbin, China; 4 Department of Cardiology, The Second Affiliated Hospital of Heilongjiang University of Chinese Medicine, Harbin, China; 5 Department of Laboratory Medicine, The Fourth Affiliated Hospital of Harbin Medical University, Harbin, China; Children's National Medical Center, Washington, United States of America

## Abstract

Intercellular adhesion molecule-1 (ICAM-1), an important immune adhesion molecule, is related to the atherosclerosis. We explored the association between the polymorphisms of the ICAM-1 gene and coronary atherosclerotic stenosis to determine whether any risk factors correlate with genetic polymorphisms in Chinese patients with coronary atherosclerosis. Using the SNaPshot assay, we examined six SNPs of rs5491, rs281428, rs281432, rs5496, rs5498 and rs281437 in 604 patients diagnosed with coronary atherosclerotic stenosis by angiography and in 468 controls. We found that AG genotype of rs5498 had higher frequency in the coronary atherosclerotic stenosis patients (41.56% to 34.19%, P = 0.017, OR = 1.368,95%CI 1.057–1.770) and that the haplotype A_rs5491_C_rs281428_G_rs281432_ had higher frequency in patients (13.8% to 12.1%, P = 0.048). When analyzing the clinical risk factors for coronary atherosclerosis, we found that the rs5498 locus was associated with the levels of apolipoprotein A (APOA) (P = 0.0002) and triglycerides (TG) (P = 0.002). Furthermore, the levels of triglycerides (TG) were also associated with rs281432 (P = 0.040). Additionally, the TT genotype of rs281437 was associated with a higher level of apolipoprotein A (APOA) (P = 0.039) and apolipoprotein B (APOB) (P = 0.003). Finally, among those with coronary atherosclerosis, we found no differences in the haplotype analysis of polymorphisms of the ICAM-1 gene from individuals with hypertension or those who smoked. According to our results, the ICAM-1 polymorphisms were associated with risk of coronary atherosclerotic stenosis in Chinese individuals.

## Introduction

Coronary heart disease, such as coronary atherosclerosis or ischemic stroke, is a common disease in the elderly population, poses a threat to public health, and can even be life-threatening. Serial angiographic studies have revealed that the plaque at the site of the culprit lesion of a future acute myocardial infarction often does not cause a stenosis that, as shown on an antecedent angiogram, is sufficiently severe to limit flow. Immune factors, particularly vascular adhesion molecules, may be play important roles in the plaque rupture and thrombosis, especially for vascular adhesion molecules [1], [2]. The accumulation of atherosclerotic plaques can be regulated by the monocyte recruitment, which is facilitated by the binding of endothelial adhesion molecules, including selectins, intercellular adhesion molecule 1 (ICAM-1) and vascular adhesion molecule 1 (VCAM-1) [3]. ICAM-1, the most common member of immunoglobulin superfamily, is typically expressed on endothelial cells and cells of the immune system. The ICAM-1 gene is located on chromosome 19 p13.2–p13.3.

Interactions between cellular adhesion molecules and their co-stimulatory receptors, such as ICAM-1 and LFA-1, can transmit signals into leukocytes and cause the migration of leukocytes into the basement membrane of the vasculature. The cellular adhesion molecule ICAM-1, which binds to LFA-1, is important for the firm adhesion of monocytes to the luminal surface of the endothelium. These monocytes can differentiate into foam cells, which participated in the processes of atherosclerotic plaque formation and inflammation in the vasculature [3]–[5]. The risk factors for atherosclerosis, such as hypertension, hyperlipidemia and low-density lipoprotein, are associated with the level of adhesion molecules, and ICAM-1 maybe considered as a prognostic factor for atherosclerosis [6], [7].

Single nucleotide polymorphisms (SNPs) of immune functional factors appears to play important roles in cardiovascular disease [8]–[12]. Studies of the association study between ICAM-1 polymorphisms and cardiovascular disease focused on two SNPs in exons, including K469E and G241R [13], [14]. Current research on the relationship between the genetic variations in the ICAM-1 gene and coronary atherosclerosis is increasingly relevant.

To investigate the relationship between SNPs of the ICAM-1 gene and the clinical indications of coronary atherosclerosis cases, we analyzed six representative SNPs in the ICAM-1 gene and performed a correlative analysis in patients with coronary atherosclerosis using clinical features collected during examinations.

## Materials and Methods

### Subjects and methods

The research group included 604 Chinese patients with coronary atherosclerosis who were recruited between 2009 and 2013. The enrolled subjects, aged 28 to 89 years (mean 63.95±10.97), were recruited from the Fourth Affiliated Hospital of Harbin Medical University. The clinical features of coronary atherosclerosis, including age, sex, hypertension, smoking history, cholesterol, blood-glucose, triglyceride, high and low density lipoprotein, were obtained from the case history of each patient. We also collected 468 normal controls, mean age 62.35±9.50 (range, 29 to 82), from the same district. The ethical board of Harbin Medical University approved the study before beginning any research and all of the participants provided written informed consent.

### Clinical definitions

All of the enrolled patients with coronary atherosclerosis were experiencing their first attack and were not receiving medication. In addition, all patients were given a definite diagnosis by coronary angiography and had clinical symptoms, such as chest pain, S-T segment change upon electrocardiogram and 64-multidetector or 320-multidetector CT examination, and the stenosis of coronary artery was more than 50%. The consensus definition was the achievement of a minimum stenosis diameter reduction of <50% with no significant coronary artery disease [15]. We divided the coronary atherosclerosis patients into two groups, medium stenosis group (50%–80% stenosis) and the severe stenosis group (>80% stenosis).

The risk factors for atherosclerosis that were analyzed included age, male gender, smoking, drinking, hyperglycaemia, hypercholesterolemia and hypertension. Smoking was defined as the individuals are smoking now or have been smoking less than one year.. Hyperglycaemia was defined as a fasting blood glucose level greater than>7.8 mmol/L, or treatment with dietary modification, insulin, or oral hypoglycemic agents. Hypertension was defined as systolic blood pressure greater than 140 mm Hg or a diastolic pressure greater than 90 mm Hg on at least three occasions, or if the patient was being treated with medication for hypertension. Hypercholesterolemia was defined as a fasting cholesterol level of more than 6.2 mmol/L or treatment with a lipid-lowering medication.

The normal controls were individuals who must satisfy three condition: (1) No typical clinical symptoms of coronary heart disease and S-T segment change on electrocardiogram (ECG). (2) The risk factors of coronary heart disease must be within normal limits, such as CHO, TG, HDL, LDL, APOA, APOB, GLU, UA and blood pressure. (3) Negative results of 64-multidetector and 320-multidetector CT examination, or treadmill exercise ECG.

### SNP selecting and genotyping

The selection of SNPs was performed with Haploview 4.0 through pairwise tagging (r^2^>0.8) and the selection of minor allele frequencies included those greater than 0.05 using Chinese (CHB) genotype data from HapMap (http://www.hapmap.org/) covering the ICAM-1 gene. Six SNPs (rs5491, rs281428, rs281432, rs5496, rs5498 and rs281437) of the ICAM-1 gene were selected as research targets.

Genomic DNA was extracted from whole blood using the Universal Genomic DNA Extraction Kit Ver. 3.0 (TaKaRa, Japan). The SNaPshot SNP assay was performed to detect the dimorphisms at the six SNP loci. The PCR primer pairs used to amplify the DNA sequences are shown in **Table S1**. PCR was performed in a 15 µl reaction mixture containing 1 µl (10 ng) of template DNA, 1 µM of each primer, 0.3 mM of each deoxynucleotide triphosphate, 3.0 mM of MgCl2, and 1 U Taq polymerase (Fermentas, USA) with 10×buffer. The PCR program consisted of an initial melting step of 3 minutes at 95°C; 11 cycles of 15 seconds at 94°C, 15 seconds at 60°C–0.5°C/cycle, and 30 seconds at 72°C; 24 cycles of 15 seconds at 94°C, 15 seconds at 54°C, and 30 seconds at 72°C; and a final elongation step of 3 minutes at 72°C. To purify the PCR products, 1 U FastAP and ExoI were mixed with 3 µl PCR product for 15 minutes at 37°C and 15 minutes at 80°C. The SNaPshot multiplex single-base extension reaction primer sequences of each SNP are also shown in **Table S1**. The extension reaction was performed in a 6 µl reaction mixture containing 1 µl of the SNaPshot Multiplex Kit (Applied Biosystems, USA), 2 µl of purified PCR products, 0.8 µM of the extension reaction primer, and 1 µl water. The PCR program was 1 minute at 96°C; 30 cycles of 10 seconds at 96°C, 5 seconds at 52°C, and 30 seconds at 60°C.

The sequencing results were analyzed with an ABI3730XL sequencer. To ensure quality control (QC), a random sample of 5% of the cases was genotyped twice by different persons, with a reproducibility of 100%.

### Statistical analysis

The data were expressed as the mean±SEM. The genotype frequencies of the SNPs were determined for the Hardy-Weinberg equilibrium (HWE) among coronary atherosclerosis cases and normal controls. The risks between SNPs and coronary atherosclerosis were estimated by odds ratio (OR) with 95% confidence interval (CI). Disease characteristics and risk factors were compared among all patients using by the chi-square test and ANOVA. The genotype frequencies of the subjects were determined using different model of inheritance (additive, dominant, recessive and homozygote comparison) and were analyzed using the chi-square test. The comparisons of the distributions of the allele, genotype and haplotype frequencies were performed using the chi-square test, and statistical significance was set at P<0.05. The statistical analyses were performed using SPSS 16.0 software.

We used Haploview 4.1 software to tag all common haplotypes and their frequencies in cases and controls. The haplotype block definitions were based on the confidence limits for a strong LD of upper = 0.85 and lower = 0.70, an upper confidence interval maximum for strong recombination of 0.85, and at least 0.8 of strong LD in informative comparisons.

## Results

### Genotyping and patient characteristics

**Figure 1:**
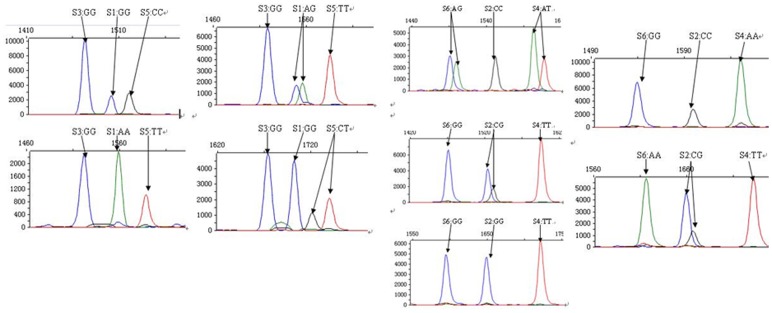
SNP genotyping of ICAM-1 gene (S1.rs281428, S2.rs281432, S3.rs5496, S4.rs5491, S5.rs5498, S6.rs281437).

Demographic and clinical characteristics are shown in **Table 1**. A total of 604 of the coronary atherosclerosis patients and 468 controls were successfully genotyped by the SNaPshot assay (**Fig. 1**). The rs5496 locus had one genotype (GG), thus we removed the rs5496 locus from our research. The other five SNPs were all consistent with the Hardy–Weinberg equilibrium.

**Table 1:** Clinical and Demographic Characteristics of Patients (n = 604) and controls (n = 468).

Variable		Mean ± S.D.	
Age (years)			
Cases		63.95±10.97	
Controls		62.35±9.50	
Sex			
Cases	Females (%)	267(44.21)	
	Males (%)	337 (55.79)	
Controls	Females(%)	199(42.52)	
	Males (%)	269(57.48)	
The clinical characteristics of patients and controls		Patients	Controls
HDL, mmol/L		1.15±0.24	1.11±0.16
LDL, mmol/L		3.00±0.96	2.65±0.57
Total CHO, mmol/L		4.97±1.18	4.59±0.68
TG, mmol/L		2.04±1.56	1.12±0.33
BMI, kg/m^2^		25.31±3.27	22.48±1.79
Systolic blood pressure (mmHg)		137.22±19.81	117.28±9.76
Diastolic blood pressure (mmHg)		81.35±11.86	78.26±10.93
APO A (g/L)		1.27±0.24	1.20±0.13
APO B (g/L)		0.96±0.32	0.84±0.19
GLU (mmol/L)		5.99±1.94	5.22±0.94
UA (umol/L)		358.49±98.41	300.97±67.09
The smoking and stenosis of coronary artery in patients			
Current or ex-smokers (%)		182 (30.32%)	
Stenosis of coronary artery (%)			
50%–80%		295 (48.84)	
More than 80%		309 (51.16)	

HDL  =  high-density lipoprotein cholesterol; LDL  =  low-density lipoprotein cholesterol; CHO =  cholesterol; TG  =  Triglyceride; BMI  =  body mass index; APO = apolipoprotein; GLU = fasting blood-glucose; UA = uric acid.

### Genotypes, alleles and haplotypes

The frequencies of the genotypes and alleles of the five SNPs of the ICAM-1 gene for both coronary atherosclerosis cancer patients and controls are shown in **Tables 2** and **Table S2**. All genotypes of the 5 SNPs were in accordance with the Hardy–Weinberg equilibrium in the breast cancer case and control groups. There was a statistically significant difference in the distribution of the genotype rs5498 when comparing coronary atherosclerosis case and control groups, we found that AG genotype had higher frequency in cases than in controls (P = 0.017, OR = 1.368 95%CI 1.057–1.770). We found no relationship among cases and controls regarding the distribution of the other four SNPs. Additionally, there was no significant difference in the distribution of the alleles of the ICAM-1 gene SNPs among controls and patients with coronary atherosclerosis (**Table S2**).

**Table 2:** The association between polymorphisms of ICAM-1 gene and coronary atherosclerosis.

SNP	Genotypes	Cases(%)	Controls(%)	P value, OR(95%CI)	Global P
rs5491	AA	531(87.91%)	405(86.54%)	reference	0.789
	AT	71(11.75%)	61(13.03%)	0.523, 0.888(0.616–1.280)	
	TT	2(0.34%)	2(0.43%)	0.581*, 0.763(0.107–5.438)	
rs281428	CC	468(77.48%)	360(76.92%)	reference	0.397
	CT	122(20.20%)	102(21.80%)	0.582, 0.920(0.684–1.238)	
	TT	14(2.32%)	6(1.28%)	0.229, 1.795(0.683–4.717)	
rs281432	CC	269(44.54%)	228(48.72%)	reference	0.386
	CG	265(43.87%)	188(40.17%)	0.175, 1.195(0.924–1.545)	
	GG	70(11.59%)	52(11.11%)	0.518, 1.141(0.765–1.702)	
rs5498	AA	305(50.50%)	266(56.84%)	reference	**0.048**
	AG	251(41.56%)	160(34.19%)	**0.017, 1.368(1.057**–**1.770)**	
	GG	48(7.94%)	42(8.97%)	0.988, 0.997(0.638–1.556)	
rs281437	CC	472(78.15%)	358(76.50%)	reference	0.597
	CT	120(19.87%)	103(22.00%)	0.414, 0.884(0.657–1.189)	
	TT	12(1.98%)	7(1.50%)	0.584, 1.300(0.507–3.336)	

* Fisher Exact test. Significant values (p<0.05) are in bold.

cases: n = 604; missing, n = 0; controls, n = 468; missing, n = 0.

### Relationship between patients' characteristics and polymorphisms of ICAM-1 gene

We analyzed the relationship between the stenosis of patients with coronary atherosclerosis and the polymorphisms of the ICAM-1 gene by chi-square test, and we found no significant difference (**Table S3**). The age- and BMI- of those patients with different the genotypes of the ICAM-1 SNPs were not significantly different by ANOVA analysis (P>0.05). There was also no significant difference among the gender compared with the various ICAM-1 SNPs using R*C chi-square (P>0.05). The age-, sex- and BMI- were shown as the mean values in **Table S4**.

Hypertension was defined as a systolic blood pressure greater than 130 mmHg or a diastolic blood pressure greater than 90 mmHg. The associations between blood pressure and ICAM-1 gene polymorphisms in patients with coronary atherosclerosis are shown in **Table 3**, We found that those with a GG genotype in rs5498 had a higher frequency of coronary atherosclerosis cases (P = 0.017, OR = 2.398, 95%CI 1.151–4.994). We also found a relationship between being a current or ex-smoker and possessing specific ICAM-1 gene polymorphisms by chi-square analysis; the TT genotype and the T allele were more frequently found in current or ex-smokers (P = 0.024 OR = 3.531, 95%CI 1.102–11.321 and P = 0.025, OR = 1.510, 95%CI 1.052–2.167, respectively) (**Table 3**).

**Table 3:** Hypertention or smoking and ICAM-1 gene polymorphisms (n = 604).

	SNPs	Genotypes and alleles	Hypertention or smoking		P value	OR(95%CI)	Global P
			Yes	No			
Hypertention	Rs5491	AA	334(88.13%)	197(87.56%)	Reference		0.922
		AT	44(11.61%)	27(12.00%)	0.879	0.691(0.557–1.601)	
		TT	1(0.26%)	1(0.44%)	0.605*	0.590(0.037–9.483)	
		A	712(93.93%)	421(93.56%)	Reference		
		T	46(6.07%)	29(6.44%)	0.794	0.938(0.580–1.516	
	Rs281428	CC	292(77.04%)	176(78.22%)	Reference		0.945
		CT	78(20.58%)	44(19.56%)	0.754	1.068(0.706–1.617)	
		TT	9(2.37%)	5(2.22%)	0.885	1.085(0.358–3.289)	
		C	662(87.34%)	396(88.00%)	Reference		
		T	96(12.66%)	54(12.00%)	0.735	1.063(0.745–1.518)	
	Rs281432	CC	171(45.12%)	98(43.56%)	Reference		0.421
		CG	160(42.22%)	105(46.67%)	0.447	0.873(0.616–1.239)	
		GG	48(12.66%)	22(9.77%)	0.436	1.250(0.713–2.194)	
		C	502(66.23%)	301(66.89%)	Reference		
		G	256(33.77%)	149(33.11%)	0.814	1.030(0.804–1.319)	
	Rs5498	AA	187(49.34%)	118(52.44%)	Reference		**0.049**
		AG	154(40.63%)	97(43.11%)	0.992	1.002(0.711–1.412)	
		GG	**38(10.03%)**	**10(4.45%)**	**0.017**	**2.398(1.151**–**4.994)**	
		A	528(69.66%)	333(74.00%)	Reference		
		G	230(30.34%)	117(26.00%)	0.107	1.240(0.955–1.610)	
	Rs281437	CC	300(79.16%)	172(76.45%)	Reference		0.386
		CT	70(18.47%)	50(22.22%)	0.291	0.803(0.534–1.208)	
		TT	9(2.37%)	3(1.33%)	0.313*	1.720(0.459–6.439)	
		C	670(88.39%)	394(87.56%)	Reference		
		T	88(11.61%)	56(12.44%)	0.665	0.924(0.646–1.321)	
Smoking	Rs5491	AA	162(89.01%)	369(87.44%)	Reference		0.672
		AT	19(10.44%)	52(12.32%)	0.518	0.832(0.477–1.453)	
		TT	1(0.55%)	1(0.24%)	0.519*	2.278(0.142–36.641)	
		A	343(94.23%)	790(93.60%)	Reference		
		T	21(5.77%)	54(6.40%)	0.678	0.896(0.533–1.506)	
	Rs281428	CC	134(73.63%)	334(79.15%)	Reference		0.266
		CT	42(23.08%)	80(18.95%)	0.213	1.309(0.856–1.999)	
		TT	6(3.29%)	8(1.90%)	0.248	1.869(0.637–5.490)	
		C	310(85.16%)	748(88.63%)	Reference		
		T	54(14.84%)	96(11.37%)	0.094	1.357(0.948–1.943)	
	Rs281432	CC	86(47.25%)	183(43.36%)	Reference		0.577
		CG	74(40.66%)	191(45.26%)	0.308	0.824(0.569–1.195)	
		GG	22(12.09%)	48(11.38%)	0.931	0.975(0.554–1.718)	
		C	246(67.58%)	557(66.00%)	Reference		
		G	118(32.42%)	287(34.00%)	0.592	0.931(0.717–1.209)	
	Rs5498	AA	96(52.75%)	209(49.53%)	Reference		0.634
		AG	74(40.66%)	177(41.94%)	0.612	0.910(0.633–1.309)	
		GG	12(6.59%)	36(8.53%)	0.365	0.726(0.362–1.456)	
		A	266(73.08%)	595(70.50%)	Reference		
		G	98(26.92%)	249(29.50%)	0.363	0.880(0.669–1.159)	
	Rs281437	CC	134(73.63%)	338(80.10%)	Reference		**0.046**
		CT	41(22.53%)	79(18.72%)	0.216	1.309(0.854–2.006)	
		TT	**7(3.84%)**	**5(1.18%)**	**0.024**	**3.531(1.102**–**11.321)**	
		C	309(84.89%)	755(89.45%)	Reference		
		T	**55(15.11%)**	**89(10.55%)**	**0.025**	**1.510(1.052**–**2.167)**	

*Fisher Exact Test.

### Association between clinical risk factors of coronary atherosclerosis and polymorphisms of ICAM-1 gene

We collected relevant clinical factors that may contribute to the stenosis of patients with coronary atherosclerosis, including total cholesterol (CHO), triglycerides (TG), high-density (HDL) and low-density lipoprotein cholesterol (LDL), apolipoprotein (APO), uric acid (UA) and fasting blood-glucose (GLU). According to our statistical analysis, patients with the GG genotype in rs281432 and rs5498 had a higher level of TG (P = 0.040 and 0.002, respectively, **Table** **4**). We found that patients with the AA genotype in rs5498 (P = 0.0002) and the TT in rs281437 (P = 0.039) had a higher level of APOA. Patients with the TT genotype in rs281437 also had a higher level of APOB (P = 0.003). We found no association between the ICAM-1 gene polymorphisms and other risk factors of coronary atherosclerotic stenosis, such as CHO, HDL, LDL, UA and GLU. The data regarding clinical risk factors were analyzed by ANOVA.

**Table 4:** Relationship between ICAM-1 polymorphisms and risk factor of coronary atherosclerosis in coronary stenosis patients.

Variable	SNPs loci	Genotype			P value
		AA	Aa	aa	
CHO, mmol/L	rs5491	4.96±1.15	4.94±1.40	6.05±0.17	0.425
	rs281428	4.96±1.17	5.00±1.19	4.73±1.41	0.728
	rs281432	4.92±1.17	5.04±1.19	4.82±1.19	0.280
	rs5498	4.95±1.20	4.96±1.18	5.08±1.03	0.777
	rs281437	4.96±1.19	4.98±1.17	5.08±1.18	0.924
TG, mmol/L	rs5491	1.98±1.46	2.35±2.11	3.66±2.37	0.060
	rs281428	1.99±1.38	2.12±1.99	2.69±2.57	0.202
	rs281432	2.08±1.60	1.89±1.30	**2.40±2.17**	**0.040**
	rs5498	2.05±1.53	1.88±1.33	**2.76±2.47**	**0.002**
	rs281437	2.02±1.53	2.14±1.77	1.58±0.36	0.447
HDL, mmol/L	rs5491	1.16±0.25	1.12±0.21	1.07±0.02	0.494
	rs281428	1.15±0.24	1.18±0.25	1.08±0.17	0.227
	rs281432	1.13±0.24	1.17±0.24	1.15±0.23	0.153
	rs5498	1.14±0.24	1.17±0.25	1.14±0.20	0.232
	rs281437	1.15±0.24	1.17±0.25	1.04±0.15	0.212
LDL, mmol/L	rs5491	2.99±0.94	2.97±1.17	3.70±0.40	0.576
	rs281428	3.00±0.97	2.98±0.93	2.89±1.55	0.911
	rs281432	2.96±0.96	3.06±0.98	2.85±0.97	0.219
	rs5498	2.99±0.95	3.03±0.97	2.85±1.03	0.577
	rs281437	3.03±0.96	2.89±0.96	2.71±1.15	0.243
APOA, g/L	rs5491	1.27±0.25	1.24±0.22	1.38±0.02	0.538
	rs281428	1.27±0.24	1.29±0.26	1.23±0.23	0.505
	rs281432	1.25±0.25	1.28±0.24	1.29±0.25	0.291
	rs5498	**1.31±0.25**	1.22±0.24	1.25±0.21	**0.0002**
	rs281437	1.28±0.25	1.22±0.23	**1.32±0.26**	**0.039**
APOB, g/L	rs5491	0.95±0.31	0.97±0.37	1.24±0.12	0.401
	rs281428	0.96±0.32	0.95±0.30	0.96±0.37	0.960
	rs281432	0.94±0.32	0.98±0.32	0.92±0.32	0.215
	rs5498	0.98±0.31	0.94±0.33	0.90±0.33	0.116
	rs281437	0.97±0.32	0.87±0.28	**1.06±0.47**	**0.003**
UA, umol/L	rs5491	356.72±97.97	369.84±105.24	383.50±64.60	0.541
	rs281428	360.22±98.67	348.86±100.73	378.70±80.65	0.390
	rs281432	362.02±92.81	354.44±107.99	359.09±83.48	0.675
	rs5498	358.35±94.52	358.73±106.91	356.39±79.86	0.989
	rs281437	358.25±97.87	355.90±103.67	386.91±86.24	0.585
GLU, mmol/L	rs5491	6.00±1.99	6.00±1.55	6.72±0.08	0.873
	rs281428	6.01±1.89	6.03±2.22	6.00±0.98	0.731
	rs281432	6.10±2.11	5.91±1.82	6.00±1.72	0.534
	rs5498	6.06±2.03	5.91±1.83	6.14±1.98	0.579
	rs281437	6.03±1.98	5.92±1.88	5.73±1.00	0.764

rs5491 A/T, rs281428 C/T, rs281432 C/G, rs5498 A/G, rs281437 C/T.

CHO =  cholesterol; TG  =  Triglyceride; HDL  =  high-density lipoprotein cholesterol; LDL  =  low-density lipoprotein cholesterol; APO = apolipoprotein; UA = uric acid; GLU = fasting blood-glucose.

The data were analyzed by ANOVA and shown as mean value.

### Haplotypes of polymorphisms of ICAM-1 gene in coronary atherosclerosis

**Figure 2:**
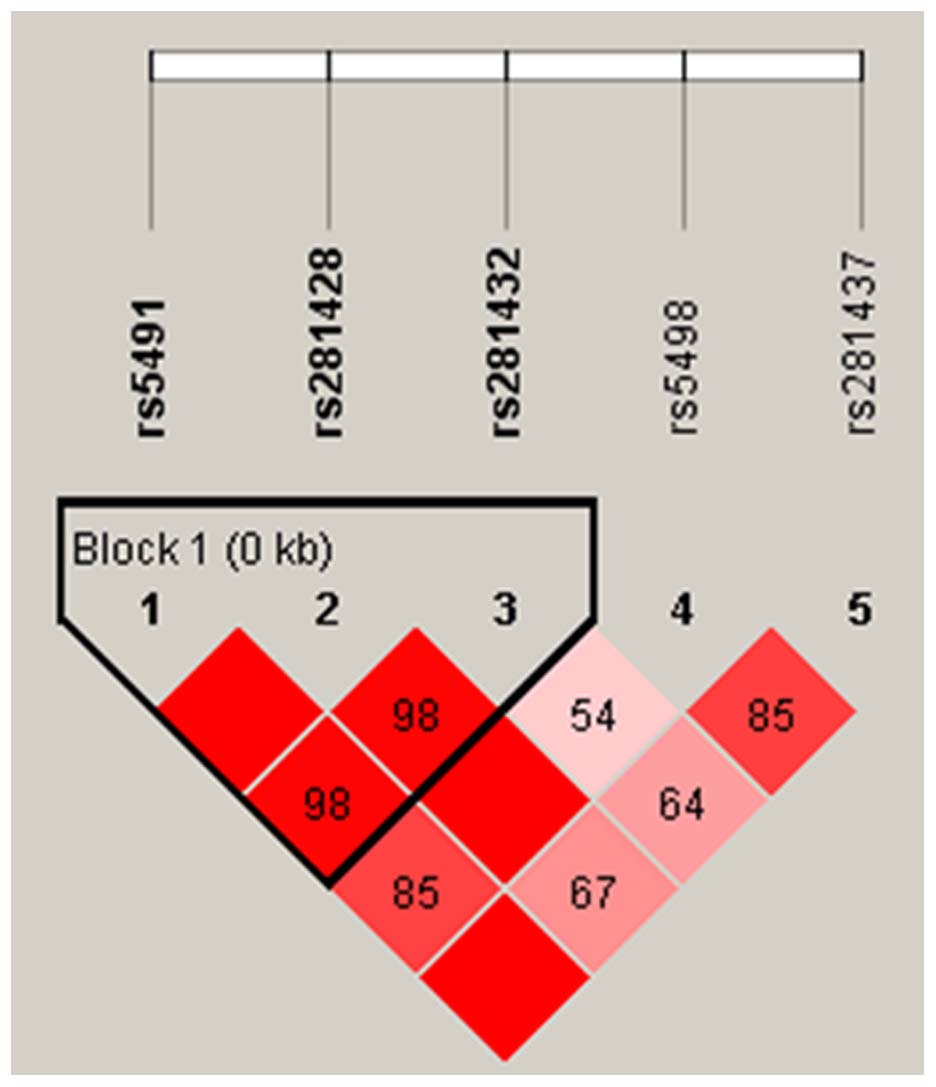
Blocks of the haplotypes in ICAM-1 gene between coronary atherosclerosis cases and controls.

We identified a haplotype block using Haploview 4.1 (**Fig. 2**). As shown in **Table** **5**, we found that there were four haplotypes that had a frequency of more than 5%, in ICAM-1 polymorphisms among the coronary atherosclerosis case and control groups. The haplotype A_rs5491_C_rs281428_C_rs281432_ had the highest frequency, but the haplotype A_rs5491_C_rs281428_G_rs281432_ had a higher frequency in cases than in controls (P = 0.0483).

**Table 5:** Haplotypes of ICAM-1 gene and risk of coronary atherosclerosis.

Haplotypes			Frequency	Cases	Controls	P value
Rs5491	Rs281428	Rs281432				
A	C	C	0.674	0.663	0.688	0.2211
A	C	G	0.138	0.151	0.121	**0.0483**
A	T	G	0.121	0.123	0.119	0.7571
T	C	G	0.065	0.061	0.070	0.4100

We analyzed the haplotypes of the ICAM-1 gene in those patients with hypertension or smoking. As shown in **Table S5**, the most frequent haplotype that appeared in cases and controls was A_rs5491_C_rs281428_C_rs281432_A_rs5498_C_rs281437_ (55.2%). We found no association between any haplotype of the ICAM-1 gene and the risk factors of hypertension or smoking in patients with coronary atherosclerosis (P>0.05).

## Discussion

Coronary atherosclerosis is a common disease in the elderly population of China, though the disease has only recently become established in China.. The attachment of circulating monocytes to vascular endothelial cells is an important link in the growth regulatory mechanisms and formation of atherosclerosis [16], [17]. Adhesion molecules can mediate, this attachment, particularly ICAM-1, which participates in the proliferation and migration of cells [18]. ICAM-1, is a pivotal immuno adhesion molecule expressed on leukocytes and platelets. As an endothelial integrin ligands, ICAM-1 mediates the adhesion and migration of leukocytes to the endothelium, and it is seldom expressed in normal coronary arteries [19]. Thus ICAM-1 might be associated with the pathology of coronary atherosclerosis, especially the soluble conformation [6], [20]. Certain risk factors play an important roles in the occurrence and development of coronary atherosclerosis, such as smoking, hypertension, aberrant levels of triglycerides, LDL and total cholesterol [21], [22].

In this study, we selected six SNPs of the ICAM-1 gene using the HapMap database, but rs5496 was excluded because it has only one genotype. The other five SNPs were located in introns, exons or the 3′-UTR. The SNPs rs5491 and rs5498 were in exons; rs281428 and rs281432 were in introns; and rs281437 was in the 3′-UTR. The polymorphism in the exon, which leads to missense mutation, can affect the protein coding functions [23]. The rs5491 (A/T, Lys-Met) and rs5498 (A/G, Lys-Glu) are missense mutations. We found that rs5498 showed a significant difference between cases and controls in our study, so this SNP may affect the structure of ICAM-1 and even the binding ability. Traditionally, introns have not been deemed as important as other genetic structures. However, in recent years, introns have been found to perform a vital role in gene transcription, RNA stability and gene processing and therefore, polymorphisms in introns can induce the aberrant splicing because of a disruption of the splice site [24], [25]. The 3'-UTR can regulate gene expression at different levels, and mediates the stability, degradation, and subcellular localization of mRNA [26]. The 3′-UTR can also be the site of microRNA-binding [27]. The genotypes of the SNPs rs281428, rs281432 and rs281437 were not significantly different among the case and control groups in our study. We also found no genetic variations of ICAM-1 that were associated with the severity of coronary atherosclerotic stenosis.

Serum total cholesterol (CHO), high-density lipoprotein (HDL) and low-density lipoprotein (LDL) levels have a high correlation with coronary atherosclerosis [28], [29]. In our research, we found no relationship between the polymorphisms of the ICAM-1 gene and patients with coronary atherosclerosis. Apolipoprotein A (APOA) was an important clinical parameter for coronary atherosclerosis. It is well-known that a decrease in apolipoprotein A is associated with the coronary atherosclerotic stenosis [30], [31]. We found that a GG genotype in the SNP rs5498 and a TT genotype in the SNP rs281437 had higher levels of APOA, and therefore, patients with a TT genotype may be less at risk for coronary atherosclerosis. A high APOB serum level may be associated with hyperlipidemia and atherosclerosis [32], and therefore, the TT genotype in rs281437 may be a risk factor for atherosclerosis cases. Individuals with a high serum level of fasting blood-glucose (GLU) and triglycerides (TG) were highly susceptible to coronary atherosclerosis [33]. In this research, we found that a GG genotype of rs281432 and rs5498 was associated with a high level of TG in coronary atherosclerosis cases (P<0.05), especially for rs5498 (P<0.01). Thus, patients with a GG genotype of rs281432 and rs5498 in the ICAM-1 gene may be more susceptible to coronary stenosis. We found no polymorphisms association in GLU of patients. In the statistical analysis of ICAM-1 gene polymorphisms and coronary atherosclerosis, we found the statistical difference (P<0.05) in the genotypes, haplotypes and risk factors. But only few of the results had significant difference (P<0.01), so the further analyis of ICAM-1 gene polymorphisms and coronary atherosclerosis should focus on more samples and statistical analysis.

To further identify the relationship between ICAM-1 gene polymorphisms and risk factors in patients with coronary atherosclerosis, including hypertension and smoking, the haplotypes of the ICAM-1 gene were analyzed by Haploview software. The haplotype block A_rs5491_C_rs281428_C_rs281432_ had the highest frequency, but the haplotype A_rs5491_C_rs281428_G_rs281432_ had a higher frequency in cases than in controls, and thus, possessing A_rs5491_C_rs281428_G_rs281432_ may be a risk factor for coronary atherosclerosis in Chinese individuals. There was no significant association between specific genotypes or alleles and hypertension or smoking in our study. The block A_rs5491_C_rs281428_C_rs281432_A_rs5498_C_rs281437_ was the most frequently observed among all haplotypes, but we detected no statistical difference in the haplotypes analysis between polymorphisms and hypertension or smoking.

In this case-control study, we found the association between polymorphisms of ICAM-1 and coronary atherosclerosis patients with coronary stenosis. However, we found that several risk factors of atherosclerosis, such as the level of TG, APOA and APOB, had a relationship with the polymorphisms of ICAM-1 in coronary atherosclerosis cases. We found no other risk factors that were associated with the ICAM-1 gene polymorphisms, including sex, BMI, CHO, LDL, GLU, UA and smoking. Thus ICAM-1 genetic variations may be a relevant influencing factor when considering the risk factors of coronary atherosclerosis.

## Supporting Information

Table S1 **The primer of each SNPs in ICAM-1 gene.**
(DOC)

Table S2 **Allele frequencies of ICAM-1 polymorphisms and their associations with coronary atherosclerosis risk.**
(DOC)

Table S3 **ICAM-1 gene polymorphisms and stenosis of coronary atherosclerosis.**
(DOC)

Table S4 **Association of ICAM-1 gene polymorphisms with patients' characteristics: Age-, Sex-, and BMI-.** rs5491 A/T, rs281428 C/T, rs281432 C/G, rs5498 A/G, rs281437 C/T. Age and BMI were shown as mean and analyzed by ANOVA. Sex were shown as male percent and analyzed by chi-square.
(DOC)

Table S5 **Association between the ICAM-1 haplotypes and Stenosis of Hypertention- and Smoking-.**
(DOC)
