# Supplementary material for: Association between the Polymorphisms in Intercellular Adhesion Molecule-1 and the Risk of Coronary Atherosclerosis: A Case-Controlled Study
Source: PLoS One. 2014 Oct 13;9(10):e109658. doi: 10.1371/journal.pone.0109658 (PMC4195684; doi:10.1371/journal.pone.0109658)
Supplement: Table S1 — The primer of each SNPs in ICAM-1 gene. (DOC) [file pone.0109658.s001.doc]

Table S1 The primer of each SNPs in ICAM-1 gene

| SNP | Primer F | Primer R | extension reaction primer |
| --- | --- | --- | --- |
| rs5491 | CTGGTGACATGCAGCACCTCC | ATTGGTTGGCTATCTTCTTGC | TTTTTTTTTTTTTTTTTTTCGGGGTCTCTATGCCCAACAAC |
| rs281428 | AGATGGAGTTTTGCTGTGTCC | GACGCAAAGAGAAATTCTCGG | CGTCTCTACTAAAAATACAAAAATTAGCC |
| rs281432 | GAAGGGTGAGGTTGGCAGAG | GACAAGGACCATAGCCAACTG | TTTTTTTTTTTTTTTATCTAATCCCTGGCCTGCTCA |
| rs5496 | CCTGAAGGTCCCATAAGGTCT | ACTCACAGAGCACATTCACGG | CCCACCTCCATGTCATCTCATC |
| rs5498 | CCTGAAGGTCCCATAAGGTCT | ACTCACAGAGCACATTCACGG | TTTTTTTTTCAGAGCACATTCACGGTCACCT |
| rs281437 | CTGGGACCATAGGCTCACAAC | GATCAGGCTGTGGCTGCTTAG | TTTTTTAAGGAAGTCTGGGCAATGTTGC |
