# Supplementary material for: Association between the Polymorphisms in Intercellular Adhesion Molecule-1 and the Risk of Coronary Atherosclerosis: A Case-Controlled Study
Source: PLoS One. 2014 Oct 13;9(10):e109658. doi: 10.1371/journal.pone.0109658 (PMC4195684; doi:10.1371/journal.pone.0109658)
Supplement: Table S2 — Allele frequencies of ICAM-1 polymorphisms and their associations with coronary atherosclerosis risk. (DOC) [file pone.0109658.s002.doc]

**Table S2. Allele frequencies of ICAM-1 polymorphisms and their associations with coronary atherosclerosis risk**

| SNPs of ICAM-1 | Alleles | NO. (%) | | OR (95% CI) | P value |
| --- | --- | --- | --- | --- | --- |
|  |  | Cases(n=604) | Controls(n=468) |  |  |
| Rs5491 | A | 1133(93.79%) | 871(93.06%) | Reference |  |
|  | T | 75(6.21%) | 65(6.94%) | 0.887 (0.629-1.251) | 0.494 |
| Rs281428 | C | 1058(87.58%) | 822(87.82%) | Reference |  |
|  | T | 150(12.42%) | 114(12.18%) | 1.022(0.788-1.326) | 0.868 |
| Rs281432 | C | 803(66.47%) | 644(68.80%) | Reference |  |
|  | G | 405(33.53%) | 292(31.20%) | 1.112(0.927-1.335) | 0.253 |
| Rs5498 | A | 861(71.27%) | 692(73.93%) | Reference |  |
|  | G | 347(28.73%) | 244(26.07%) | 1.143(0.943-1.385) | 0.172 |
| Rs281437 | C | 1064(88.08%) | 829(87.63%) | Reference |  |
|  | T | 144(11.92%) | 117(12.37%) | 0.947(0.730-1.229) | 0.684 |
