# Supplementary material for: Association between the Polymorphisms in Intercellular Adhesion Molecule-1 and the Risk of Coronary Atherosclerosis: A Case-Controlled Study
Source: PLoS One. 2014 Oct 13;9(10):e109658. doi: 10.1371/journal.pone.0109658 (PMC4195684; doi:10.1371/journal.pone.0109658)
Supplement: Table S3 — ICAM-1 gene polymorphisms and stenosis of coronary atherosclerosis. (DOC) [file pone.0109658.s003.doc]

**Table S3. ICAM-1 gene polymorphisms and stenosis of coronary atherosclerosis**

| SNPs loci | Genotypes and alleles | stenosis of coronary artery (%) | | P value | OR(95%CI) | Global P |
| --- | --- | --- | --- | --- | --- | --- |
|  |  | Medium(50%-80%) | Severe (> 80%) |  |  |  |
| Rs5491  exon | AA | 263(89.15%) | 268(86.73%) | Reference |  | 0.649 |
|  | AT | 31(10.51%) | 40(12.94%) | 0.353 | 0.790(0.480-1.301) |  |
|  | TT | 1(0.34%) | 1(0.33%) | 0.746* | 1.019(0.063-16.377) |  |
|  | A | 557(94.41%) | 576(93.20%) | Reference |  |  |
|  | T | 33(5.59%) | 42(6.80%) | 0.386 | 0.813(0.508-1.301) |  |
| rs281428  intron | CC | 226(76.61%) | 242(78.32%) | Reference |  | 0.880 |
|  | CT | 62(21.02%) | 60(19.42%) | 0.619 | 1.106(0.743-1.648) |  |
|  | TT | 7(2.37%) | 7(2.26%) | 0.900 | 1.071(0.370-3.101) |  |
|  | C | 514(87.12%) | 544(88.03%) | Reference |  |  |
|  | T | 76(12.88%) | 74(11.97%) | 0.633 | 1.087(0.772-1.530) |  |
| rs281432  intron | CC | 133(45.08%) | 136(44.01%) | Reference |  | 0.565 |
|  | CG | 132(44.75) | 133(43.04%) | 0.932 | 1.015(0.723-1.425) |  |
|  | GG | 30(10.17%) | 40(12.95%) | 0.326 | 0.767(0.451-1.303) |  |
|  | C | 398(67.46%) | 405(65.53%) | Reference |  |  |
|  | G | 192(32.54%) | 213(34.47%) | 0.479 | 0.917(0.722-1.165) |  |
| rs5498  exon | AA | 155(52.54%) | 150(48.54%) | Reference |  | 0.333 |
|  | AG | 114(38.64%) | 137(44.34%) | 0.205 | 0.805(0.576-1.126) |  |
|  | GG | 26(8.82%) | 22(7.12%) | 0.521 | 1.144(0.621-2.106) |  |
|  | A | 424(71.86%) | 437(70.71%) | Reference |  |  |
|  | G | 166(28.14%) | 181(29.29%) | 0.658 | 0.945(0.737-1.213) |  |
| Rs281437  3’-UTR | CC | 227(76.95%) | 245(79.29%) | Reference |  | 0.428 |
|  | CT | 60(20.34%) | 60(19.42%) | 0.709 | 1.079(0.723-1.611) |  |
|  | TT | 8(2.71%) | 4(1.29%) | 0.164* | 2.159(0.641-7.266) |  |
|  | C | 514(87.12%) | 550(89.00%) | Reference |  |  |
|  | T | 76(12.88%) | 68(11.00%) | 0.314 | 1.196(0.844-1.695) |  |

*Fisher exact test
