# Supplementary material for: Association between the Polymorphisms in Intercellular Adhesion Molecule-1 and the Risk of Coronary Atherosclerosis: A Case-Controlled Study
Source: PLoS One. 2014 Oct 13;9(10):e109658. doi: 10.1371/journal.pone.0109658 (PMC4195684; doi:10.1371/journal.pone.0109658)
Supplement: Table S4 — Association of ICAM-1 gene polymorphisms with patients' characteristics: Age-, Sex-, and BMI-. rs5491 A/T, rs281428 C/T, rs281432 C/G, rs5498 A/G, rs281437 C/T. Age and BMI were shown as mean and analyzed by ANOVA. Sex were shown as male percent and analyzed by chi-square. (DOC) [file pone.0109658.s004.doc]

**Table S4. Association of ICAM-1 gene polymorphisms with patients’ characteristics**: Age-, Sex-, and BMI-.

| Variable | SNPs loci | genotype | | | P value |
| --- | --- | --- | --- | --- | --- |
|  |  | AA | Aa | aa |  |
| Age,y | rs5491 | 63.67±11.10 | 65.97±10.01 | 64.50±21.92 | 0.255 |
|  | rs281428 | 64.27±11.04 | 62.91±11.18 | 62.00±7.84 | 0.381 |
|  | rs281432 | 64.00±11.13 | 63.61±10.83 | 65.01±11.29 | 0.635 |
|  | rs5498 | 64.05±11.00 | 64.26±10.76 | 61.65±12.24 | 0.313 |
|  | rs281437 | 64.15±10.73 | 63.47±11.77 | 60.75±14.23 | 0.498 |
| Male, % | rs5491 | 56.31 | 50.70 | 100.00 | 0.318* |
|  | rs281428 | 55.77 | 54.10 | 71.43 | 0.465 |
|  | rs281432 | 58.36 | 53.96 | 52.86 | 0.515 |
|  | rs5498 | 57.70 | 52.59 | 60.42 | 0.210 |
|  | rs281437 | 55.51 | 55.83 | 66.67 | 0.744 |
| BMI, kg/m^2^ | rs5491 | 25.34±3.30 | 25.02±3.11 | 27.00±0.00 | 0.568 |
|  | rs281428 | 25.25±3.28 | 25.55±3.33 | 25.04±2.26 | 0.632 |
|  | rs281432 | 25.38±3.47 | 25.10±3.18 | 25.78±2.79 | 0.268 |
|  | rs5498 | 25.34±3.07 | 25.40±3.64 | 24.55±2.35 | 0.249 |
|  | rs281437 | 25.43±3.35 | 24.84±3.04 | 24.98±1.92 | 0.196 |

rs5491 A/T, rs281428 C/T, rs281432 C/G, rs5498 A/G, rs281437 C/T.

Age and BMI were shown as mean and analyzed by ANOVA. Sex were shown as male percent and analyzed by chi-square.

* Fisher exact test
