# Supplementary material for: Association between the Polymorphisms in Intercellular Adhesion Molecule-1 and the Risk of Coronary Atherosclerosis: A Case-Controlled Study
Source: PLoS One. 2014 Oct 13;9(10):e109658. doi: 10.1371/journal.pone.0109658 (PMC4195684; doi:10.1371/journal.pone.0109658)
Supplement: Table S5 — Association between the ICAM-1 haplotypes and Stenosis of Hypertention- and Smoking-. (DOC) [file pone.0109658.s005.doc]

**Table S5. Association between the ICAM-1 haplotypes and Stenosis of** Hypertention- and Smoking-.

| Haplotypes (frequency ≥5%) | | | | | Frequency | Hypertention  P value | smoking  P value |
| --- | --- | --- | --- | --- | --- | --- | --- |
| Rs5491 | rs281428 | rs281432 | rs5498 | rs281437 |
| A | C | C | A | C | 0.552 | 0.7184 | 0.9453 |
| A | C | G | G | C | 0.137 | 0.6651 | 0.2714 |
| A | T | G | A | T | 0.091 | 0.7602 | 0.0895 |
| A | C | C | G | C | 0.090 | 0.3058 | 0.6756 |
| T | C | G | G | C | 0.055 | 0.9110 | 0.8561 |
